# AnyFace++: Deep Multi-Task, Multi-Domain Learning for Efficient Face AI

**DOI:** 10.3390/s24185993

**Published:** 2024-09-15

**Authors:** Tomiris Rakhimzhanova, Askat Kuzdeuov, Huseyin Atakan Varol

**Affiliations:** Institute of Smart Systems and Artificial Intelligence, Nazarbayev University, Astana 010000, Kazakhstan; tomiris.khalimova@nu.edu.kz (T.R.); askat.kuzdeuov@nu.edu.kz (A.K.)

**Keywords:** multi-task learning, multi-domain learning, face detection, facial landmark detection, age estimation, gender identification, emotion recognition, YOLOv8

## Abstract

Accurate face detection and subsequent localization of facial landmarks are mandatory steps in many computer vision applications, such as emotion recognition, age estimation, and gender identification. Thanks to advancements in deep learning, numerous facial applications have been developed for human faces. However, most have to employ multiple models to accomplish several tasks simultaneously. As a result, they require more memory usage and increased inference time. Also, less attention is paid to other domains, such as animals and cartoon characters. To address these challenges, we propose an input-agnostic face model, AnyFace++, to perform multiple face-related tasks concurrently. The tasks are face detection and prediction of facial landmarks for human, animal, and cartoon faces, including age estimation, gender classification, and emotion recognition for human faces. We trained the model using deep multi-task, multi-domain learning with a heterogeneous cost function. The experimental results demonstrate that AnyFace++ generates outcomes comparable to cutting-edge models designed for specific domains.

## 1. Introduction

The development of the large-scale ImageNet [1] dataset and advancements in Graphics Processing Units (GPUs) have paved the way for training a noteworthy convolutional neural network (CNN), AlexNet [2], which surpassed all preceding traditional machine learning methods in the ILSVRC-2012 image classification competition. These technological advancements have triggered a profound growth of artificial intelligence (AI) in numerous areas such as computer vision [3], speech processing [4], natural language processing (NLP) [5], and game playing [6].

The introduction of AlexNet launched a big race on training deeper neural networks with an ever-increasing number of parameters and complexity to achieve state-of-the-art (SOTA) performance on benchmark datasets [7]. Most research papers have focused on improving the accuracy of the models rather than their efficiency [8]. As a result, the computational resources required to train deep learning (DL) models have been increasing with a doubling time of around six months [9], surpassing the rate of Moore’s Law by a significant margin. However, training and running of DL models have a significant environmental impact due to carbon emissions [10].

In 2017, the transformer neural network architecture was introduced [11]. The training of transformer-based models with hundreds of millions of parameters on large-scale datasets has led to the emergence of foundational models with zero-shot capability, such as ViT [12], Whisper [13], GPT-3 [14], and BERT [15]. The development and deployment of foundational models incur significant costs attributed to the need for high-performance computing (HPC) and an enormous environmental impact due to carbon emissions [16]. For instance, the carbon emissions from training BERT are equivalent to those produced by a cross-country flight in the United States of America [17].

The reliance on HPC restricts the accessibility of SOTA models to academia, small companies, and startups. In this regard, various methods of developing efficient DL models have appeared. For instance, Tiny Machine Learning (TinyML) is a rapidly growing concept in edge computing that integrates embedded systems (including hardware and software) with machine learning. The objective is to provide ultra-low-power, cost-effective, and secure machine learning inference capabilities to battery-powered smart devices [18]. At present, owing to memory constraints, TinyML is primarily employed to address simple tasks such as keyword spotting (e.g., “Alexa”, “Ok Google”, and “Hey Siri”) and image classification [19].

Model compression is another approach for reducing device storage requirements, speeding up model inference, simplifying model topology, and cutting down on training expenses while enhancing model deployment. The main strategies for model compression are pruning, parameter quantization, low-rank decomposition, and knowledge distillation [20]. However, these methods have inherent limitations, such as reduced accuracy and stability after compression.

Multi-task learning (MTL) is a specialized branch of machine learning that involves learning multiple tasks concurrently by a single model [21]. This approach benefits from enhanced data efficiency, overfitting reduction due to shared representations, and expedited learning by making use of auxiliary information. Multi-domain learning (MDL) is the task of training a model across multiple overlapping but non-identical domains [22]. Training a model for both MTL and MDL tasks enables the compression of information from a range of sources into a single backbone, thus improving the efficiency of the model [23,24].

In this study, we propose a deep multi-task, multi-domain method for computer vision-based efficient face analysis. Within the last decade, considerable advancements have been made in computer vision applications for human faces, such as face detection and recognition, facial emotion recognition, age detection, race identification, and gender recognition [25]. This impressive success can be attributed to the advancements in DL and the availability of large-scale datasets. However, other domains like animals and cartoon characters are less represented.

Animal face analysis can be used for identification, disease control, production management, and ownership determination. Notably, contactless visual biometric solutions are preferable over the invasive methods [26]. Cartoon faces represent another domain with a high prevalence of various facial expressions. With the growing amount of cartoon-style content in media, the need for computer vision-based tools to address face analysis tasks is also increasing. For example, face detection in comic books can be used for subsequent analysis of facial expressions [27].

In this work, we present AnyFace++, a comprehensive multi-task, multi-domain model for efficient face AI. AnyFace++ is designed to detect faces and facial landmarks in humans, animals, and cartoons in the visual and thermal domains. Also, the model predicts the domain of the detected face, such as human, animal, and cartoon. Additionally, the model predicts age, emotions, and gender in human faces. The experimental results showed that the model can generalize across all domains and tasks, delivering results on par with the SOTA models designed specifically for each task and domain. As a result, AnyFace++ can replace several single-task models developed for human, animal, and cartoon faces. We have made the source code and pre-trained models to bolster research in this area.

The rest of the paper is structured as follows: Section 2 highlights related works. In Section 3, we provide the model architecture of AnyFace++ and our multi-task loss function. We introduce employed datasets and provide training settings in Section 4. Section 5 discusses the experimental results. Finally, we conclude our work in Section 6.

## 2. Related Works

Deep multi-task learning (DMTL) is extensively used in various face applications as this method aims to use the synergy that exists among related tasks. One of the popular DMTL methods is joint face detection and facial landmark localization [28,29,30]. In this case, the models concurrently carry out tasks such as face classification, regression of facial bounding boxes, and regression of facial landmarks. The research demonstrated that using facial landmarks as an additional supervision signal significantly improves the detection of small faces [30,31]. A multi-task deep CNN was proposed to simultaneously learn face classification, face pose estimation, and facial landmark localization [32]. The results demonstrated that MTL improves the detection of challenging faces under various pose, illumination, and expression conditions. DMTL was proposed to simultaneously learn facial landmark detection, head pose estimation, and facial attribute recognition [33]. The proposed learning method was efficient in detecting faces with severe occlusion and pose variation.

DMTL is also extensively employed for recognizing facial expressions and attributes such as age, gender, and ethnicity. For instance, a multi-task cascaded CNN for joint face detection and facial expression recognition was proposed to use the inherent correlation between them [34]. A Hierarchical Multi-Task Network (HMTNet) was presented for the simultaneous recognition of gender, race, and facial attractiveness [35]. The results showed that multi-task joint training in HMTNet increases the performance in all three tasks. A multi-instance and multi-scale enhanced multi-task random forest approach was suggested for simultaneously processing classifications for age and gender [36]. The study showed that the gender of a face significantly affects the classification of face age. A deep multi-source, multi-task learning framework for smile detection, emotion recognition, and gender classification was presented in [37]. The research demonstrated that joint learning significantly benefits tasks with limited data by leveraging other tasks with more extensive data.

There has also been research into DMTL aimed at developing efficient applications for embedded devices with limited computational capacity and memory. For instance, a lightweight multi-task neural network for facial expression and attribute recognition (age, gender, and ethnicity) was presented in [38]. The experimental results demonstrated that the proposed method achieves near state-of-the-art results on the benchmark dataset. Likewise, MTL was suggested for computationally efficient recognition of gender, age, ethnicity, and emotion on the edge [39]. The presented solution exhibited its efficiency in terms of accuracy, processing time, and memory usage compared to the single-task CNN. A lightweight multi-task CNN was proposed for simultaneous age and gender classification in mobile devices [40].

Only a limited number of works are available on AI for animals. DL-based models were developed for the detection of faces in cattle [41], sheep [42], and mice [43]. DL-based methods were proposed for the classification of the emotional states of dogs based on their facial expressions [44,45]. A pain recognition in facial images of domestic short-haired cats using CNN and facial landmark-based methods was proposed in [46]. A large-scale, hierarchical dataset of animal faces, AnimalWeb, was presented in [47]. The dataset can be employed for DMTL that combines facial landmark localization, face detection, and fine-grained face recognition.

Cartoon faces are also a low-resourced domain in face AI. Several works can be found in the literature. For instance, there are studies on face detection models for the characters of manga [48], cartoons [49], and comics [50]. Face recognition for cartoon faces can enhance search engines [49,51] and improve cartoon movie recommendation systems [52]. Facial expression recognition in cartoon faces can be used for parental control [53], and it can help professionals, like animators, classify and label cartoon faces for future projects [54].

In our previous work, we developed an input-agnostic model called AnyFace [55]. AnyFace was based on the architecture of the YOLO5Face face detection model [28]. The model was trained to detect faces and facial landmarks in different domains, such as humans, animals, and cartoon characters. Although AnyFace demonstrated strong performance in these areas, it does not differentiate between the types of faces nor does it predict additional facial attributes such as age, gender, and emotion. In contrast, AnyFace++ extends these capabilities by identifying whether a face belongs to a human, animal, or cartoon character. Also, the model predicts attributes such as gender, age, and emotions for human faces.

No studies have concentrated on using multi-task, multi-domain learning to concurrently accomplish multiple tasks, such as face detection, facial landmark detection, age detection, gender identification, and emotion recognition for human, animal, and cartoon faces. The reason is that most of the datasets cover a portion of these tasks. To tackle this problem, we propose a multi-task, multi-domain learning method using a heterogeneous cost function that takes into account sparsely-labeled data.

## 3. Methodology

### 3.1. AnyFace++

In this study, we adapted the architecture of the YOLOv8 object detection model [56]. YOLOv8 is one of the latest variants of the YOLO (You Look Only Once) [57] object detection model. We opted for YOLOv8 for two primary reasons: speed and accuracy. As a one-stage object detector, YOLOv8 delivers speedy outcomes while achieving top-tier performance on the COCO benchmark dataset [58]. Also, YOLOv8 offers a number of model sizes including nano (n), small (s), medium (m), large (l), and extra large (xl). The YOLOv8 object detection model was designed to detect and classify objects of 80 classes in the COCO dataset [58]. The model was trained using the sum of Complete Intersection over Union (CIoU) [59] and Distribution Focal Loss (DFL) [60] loss functions for bounding-box regression and a cross-entropy loss for the recognition of 80 different objects. DFL predicts the possible distribution of box offsets instead of directly projecting the box coordinates, assisting in interpreting the ambiguity of the box location. CIoU is used to calculate the disparity between the predicted bounding boxes and the actual ones. The overlap area, the distance between box centers, and the aspect ratio are considered in its computation, offering a more thorough evaluation than the conventional IoU.

**Figure 1:**
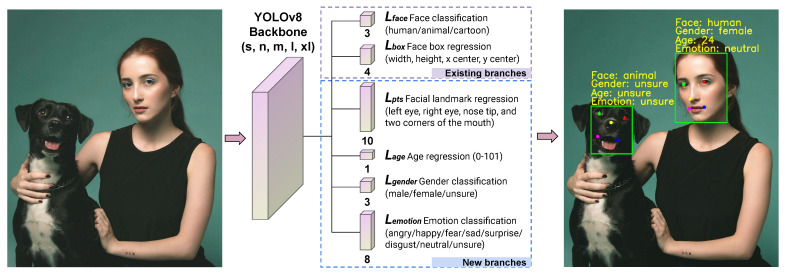
The AnyFace++ network architecture is built on the YOLOv8 backbone network, includes its two existing output layers (object classification and bounding box regression) and also introduces new output layers (facial landmark regression, age regression, gender classification, and emotion classification).

We modified the head of YOLOv8 by inserting new linear layers for our tasks. The simplified architecture of the modified network is shown in Figure 1. We used the existing classification layer of YOLOv8 for face classification and the bounding box regression layer for facial bounding boxes. We inserted new linear layers for the regression of facial landmarks and age. Also, we added new layers for the classification of gender and emotion. We kept the backbone network of YOLOv8 unchanged. In this way, we could train n, s, m, l, and xl models of YOLOv8 by attaching the new head.

The face classification layer outputs predictions for three categories: human face, animal face, and cartoon face. The model employs a categorical cross-entropy loss function for optimization. We denoted this loss function as $L_{face}$. We used the default CIoU and DLF loss functions to optimize the detection of facial bounding boxes. We denoted the sum of these two loss functions as $L_{box}$. The bounding box is represented by its width, height, and the x and y coordinates of its center.

The linear layer for the regression of facial landmarks outputs the x and y coordinates of five points: the left eye, right eye, nose tip, left corner of the mouth, and right corner of the mouth. We employed the Wing loss function [61] to optimize the regression of facial landmarks. This loss function is widely used for facial landmarks [28,62]. We notated this loss function as $L_{pts}$.

The next linear regression layer is for age estimation. We employed the mean squared error (MSE) loss function for optimization. We denoted this loss function as $L_{age}$. The model estimates age within a range of 0 and 101. We set the age as 101 for animal and cartoon faces. The reason is that age datasets are not available in these domains.

The linear layer for gender classification outputs predictions for three categories: male, female, and unsure. The “unsure” category was assigned for animal and cartoon faces due to the lack of gender datasets in these domains. We employed a categorical cross-entropy loss function for optimization. We denoted it as $L_{gender}$.

The emotion classification layer issues predictions for eight facial expressions: angry, happy, fear, sad, surprise, disgust, neutral, and unsure. The first seven emotions were chosen because they are most commonly featured in facial expression datasets. The last “unsure” class was assigned for animal and cartoon faces, since there are no relevant datasets for this task. The emotion classification output was optimized using a categorical cross-entropy loss function $L_{emotion}$.

### 3.2. Heterogeneous Loss Function

The ground-truth labels are required to compute the loss functions during the training stage. However, no dataset has all the labels for our tasks. Therefore, we assume that ground-truth labels for all tasks will not be available during the training. However, ground-truth facial bounding boxes with their corresponding face type (human, animal, cartoon) are mandatory as $L_{pts}$, $L_{age}$, $L_{gender}$, and $L_{emotion}$ are computed only for the correctly detected faces. To handle the missing labels in computing the total loss function, $L_{total}$, we propose to use a heterogeneous loss function. Consider we have *n* number of tasks to learn in a supervised manner. We can define these tasks as $T=[t_{1},t_{2},\cdots ,t_{n}]$ and their corresponding loss functions as $L=[l_{1},l_{2},\cdots ,l_{n}]$. Then, we define a state vector $S=[s_{1},s_{2},\cdots ,s_{n}]$ that represents the availability of labels for each task. If the label for the task $t_{i}$ is available, then $s_{i}=1$, otherwise, $s_{i}=0$. The total loss function is computed as:

(1) $$L_{total}=LS^{T}=l_{1}s_{1}+l_{2}s_{2}+\cdots +l_{n}s_{n}$$

In this way, the total loss is computed only for the task with the available labels. In our case, the total loss function can be defined as:

(2) $$\begin{array}{c} L_{total}=LS^{T}=L_{face}S_{face}+L_{box}S_{box}+L_{pts}S_{pts}+\\ L_{gender}S_{gender}+L_{age}S_{age}+L_{emotion}S_{emotion} \end{array}$$

However, these loss functions have different scales. For instance, $L_{face}$, $L_{gender}$, and $L_{emotion}$ are categorical cross-entropy; $L_{age}$ is MSE; $L_{pts}$ is Wing; and $L_{box}$ is the sum of CIoU and DFL. Therefore, we normalized them as follows:

(3) $$\begin{array}{c} L_{total}=LS^{T}=L_{face}S_{face}+\lambda_{1}L_{box}S_{box}+\lambda_{2}L_{pts}S_{pts}+\\ L_{gender}S_{gender}+\lambda_{3}L_{age}S_{age}+L_{emotion}S_{emotion} \end{array}$$

where $\lambda_{1}$, $\lambda_{2}$, and $\lambda_{3}$ are trainable parameters.

## 4. Experiments

### 4.1. Datasets

We utilized open-source benchmark face datasets, as presented in Table 1, to train our model for age, emotion, and gender classification tasks and face and facial landmark detection. Most of the available datasets were developed for human faces. Moreover, there are no extensive datasets for animal and cartoon faces that cover emotion, gender, and age.

For animal faces, we used AnimalWeb [47], a large-scale, hierarchical dataset of animal faces. The dataset comprises about 22,400 faces from 350 distinct species and 21 animal orders spanning various levels of biological taxonomy. The dataset provides annotations for nine-point facial landmarks. We converted the facial landmarks into the five-point configuration for our task. Also, we generated facial bounding boxes using the coordinates of the facial landmarks. Annotations for age, gender, and emotions are not available.

For cartoon faces, we employed a large-scale iCartoonFace dataset [76], which contains multiple styles. The dataset contains 50,000 training images (91,163 labeled bounding boxes) and 10,000 testing images (18,647 labeled bounding boxes). However, the dataset does not have annotations for facial landmarks, age, gender, and emotions.

Fifteen datasets were utilized for human facial analysis, with varying annotations: three included emotion labels, nine had gender labels, twelve featured age labels, and six provided facial landmarks. These datasets encompassed bounding box coordinates either directly provided by the authors or through image cropping, where we added bounding box coordinates as the size of the image. For datasets lacking this information, we incorporated it using a face detection model. In addition, Adience, RAF-DB, and FairFace datasets are characterized by age representations in range format, so an additional preprocessing step was necessitated, whereby the mean value in each age range was carefully selected and subsequently used as a facial label.

The Facial Expression Recognition (FER) dataset [63] consists of 35,852 grayscale facial images cropped to a resolution of 48 × 48 pixels. The dataset is divided into 28,709 images for training and 7178 for testing. The dataset provides annotations for the seven facial expressions considered in this work.

AffectNet [64] is a large-scale facial expression dataset with around 0.4 million images manually labeled for the presence of eight emotions. In our study, we used 287,401 images that represent the seven emotions. The images have a resolution of 224 × 224 pixels. Additionally, the dataset provides labels for facial landmarks. For our study, we adopted the same data split as in [77], with approximately 280 thousand images for training, 3500 for testing (500 images per category), and the remaining images for validation.

The Real-world Affective Faces Database (RAF-DB) [74] is a manually labeled large-scale dataset with labels for emotion, age, gender, bounding box, and facial landmarks. In our study, we used a subset of the dataset that contains 15,339 single-label images for emotion recognition. We used 12,271 images for training and 3068 images for testing.

The IMDB dataset [65,78] was constructed using images of celebrities from Wikipedia, accompanied by indications of their biological age and gender. The dataset contains images without faces. Thus, we filtered the dataset using the method from [79]. As a result, we obtained 285,949 images after filtering. We allocated 200,166 images for training, 57,189 for validation, and 28,594 for testing.

The AgeDB [70] dataset contains faces of famous people such as politicians, actors, writers, and others collected from the Internet. The dataset provides labels for age and gender. The dataset consists of 16,488 images with an average of 29 images per subject.

UTKFace is a large-scale dataset with labels for age and gender [66]. It encompasses over 20,000 face images captured under various poses and environmental conditions. The dataset provides 68-point facial landmarks. We converted them into five-point facial landmarks. To ensure comparability with the previous study [80], we employed the same test that consists of 3287 images. The rest of the dataset was used for training.

The Adience dataset [67] contains 26,580 images. The dataset includes both aligned and non-aligned versions captured using smartphone devices. In this work, we specifically utilized the aligned portion to prevent duplication, resulting in 13,023 images.

The FairFace dataset [71] has 108,501 cropped images, balanced across seven races and labeled with gender and age. Of these, 97,698 images are open-source and standardized to a resolution of 224 × 224 pixels. We used the default training (86,744 images) and testing (10,954 images) sets.

The Unified Age and Gender Dataset (UAGD) [72] was intentionally created to mitigate problems associated with unbalanced distributions in gender in each age group. The dataset comprises 11,852 images, each annotated with gender and age labels, and encompasses a range of resolutions and lighting conditions.

To increase the diversity of Asian faces, we employed the AFAD dataset [69]. It contains cropped RGB images with gender and age labels. We utilized 59,344 images in total. We split the dataset as in [69], where 80% of the images were selected randomly for training and the remaining 20% for testing.

We also used the Mega-Age [68] dataset and its alternative version with only Asian faces, MegaAge Asian [68]. The Mega-Age dataset comprises 41,941 images, while MegaAge Asian includes 43,945 images. Both datasets have images with a resolution of 178 × 218 pixels. We automatically labeled the dataset with facial bounding boxes using our previous AnyFace model [55]. The datasets include images of people from childhood to old age, making them valuable for studying age progression and facial detection at different stages of life.

The FG-NET dataset [73] offers annotations for bounding boxes, 68-point facial landmarks, which were subsequently transformed to 5-point landmarks, and precise age labels. In our research, we utilized only 1002 publicly available images at different resolutions, as the original website does not provide access to the complete dataset.

Wider Face [75] is a benchmark dataset for human face detection. The dataset contains 32,203 images with 393,703 labeled faces in diverse real-world environments. The dataset provides 12,880 images (159,424 faces) in the training set, 3226 images (39,798 faces) in the validation set, and 16,097 images (194,571 faces) in the test set. For human face detection in the thermal domain, we utilized the TFW dataset [31]. The dataset was collected in indoor and outdoor settings. It provides labels for bounding boxes and 5-point facial landmarks. The dataset contains 9982 thermal images with 16,509 labeled faces.

Overall, we employed 846,993 images and 1,041,407 faces, covering human, animal, and cartoon domains.

### 4.2. Training Details

Considering the size of the dataset and our computational resources, we trained a medium version of AnyFace++ in this work. However, anyone interested can train other versions (nano, small, large, and x-large) as we released the source code with training instructions. The medium version of the original YOLOv8 model has 25.9 M parameters and 78.9 Giga Floating Point Operations (GFLOPs). After modification, the medium version of AnyFace++ has 26.6 M parameters and 163.9 GFLOPs. The number of floating point operations doubled due to the addition of the new fully connected layers.

We trained the model from scratch on two A100-SXM4-40GB GPUs installed on an NVIDIA DGX server. We used image augmentation methods provided in the original YOLOv8 model, such as translation, scaling, shearing, horizontal flipping, and mosaic augmentation. We used Stochastic Gradient Descent (SGD) algorithm for optimization with the initial learning rate of 0.01. The model was trained for 200 epochs with a maximum batch size of 52. The total training time was about 234 h.

## 5. Results and Discussion

### 5.1. Face Detection

The $AP_{50}$ scores on the validation and test set of Wider Face are given in Table 2. On the validation set, AnyFace++ achieved 92.9% on easy, 91.2% on medium, and 83.8% on the hard set, respectively. On the test set, the model achieved 91.6% on easy, 90.1% on medium, and 82.9% on the hard set, respectively. Our results are quite low compared to the other models. One of the reasons is that RetinaFace [30] and YOLO5Face [28] were trained only on human faces. Moreover, these models were designed to perform only two tasks (face and facial landmark detection). Regarding AnyFace [55], it was trained on human, animal, and cartoon faces. However, it also performs only face detection and facial landmarks prediction. In our case, AnyFace++ is designed to perform more tasks, which makes optimization more complicated. Also, our model has a smaller number of parameters (26.6 M) compared to other models.

**Figure 2:**
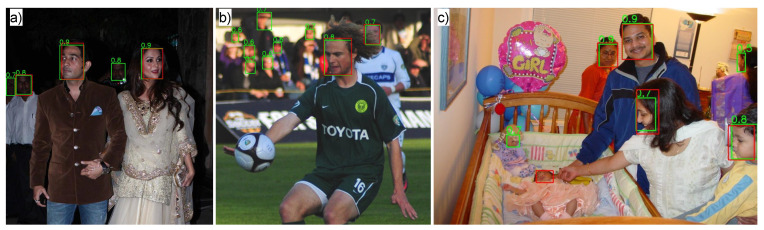
Examples of unlabeled faces in the validation set of Wider Face, detected by AnyFace++. The red bounding boxes are ground truth. The green bounding boxes with confidence scores are predictions: (**a**) dark faces, (**b**) blurry faces, and (**c**) a toy face.

We visually checked the model’s predictions on some images from the validation set of Wider Face. We found that AnyFace++ detects unlabeled faces with high confidence scores in many cases. For instance, the model detected unlabeled dark faces as shown in Figure 2a. Also, the model successfully detected many unlabeled blurry faces, as shown in Figure 2b. We also noticed the model detects toy faces as human faces (see Figure 2c). These predictions are considered as false positives during the evaluation, decreasing the overall score.

The face detection results for animal, cartoon, and thermal human faces are shown in Table 3. AnyFace++ attained an accuracy of 93.9% on animal faces, slightly surpassing the performance of the AnyFace model. When tested on the iCartoonFace dataset, AnyFace++ achieved a score of 90.8%, which is a bit lower compared to AnyFace, but similar to ACFD (90.9%) [81] and RetinaFace (91.0%) [76], both of which were trained solely on cartoon faces. For thermal human faces, AnyFace++ improved the baseline result for the outdoor dataset from 97.3% [31] to 99.3%. This outcome is also similar to the result obtained by AnyFace (99.5%). As for the indoor set of TFW, AnyFace++ achieved a perfect score of 100.0%, which is the same as other models.

### 5.2. Facial Landmark Detection

To evaluate the performance of AnyFace++ in detecting facial landmarks in thermal images, we employed the Normalized Mean Error (NME) metric, as used in TFW [31]. This metric normalizes the distance between the predicted and ground-truth facial landmarks, following the equation:

(4) $$NME=\frac{1}{N}\sum_{i=1}^{N}\frac{\left|\right|\hat{l_{i}}-l_{i}\left|\right|}{K\times D_{i}}$$

In this equation, $\left|\right|\hat{l_{i}}-l_{i}\left|\right|$ represents the Euclidean distance between the coordinates of the ground-truth ($\hat{l_{i}}$) and predicted ($l_{i}$) landmarks. *K* denotes the number of facial landmarks (five in our case), $D_{i}$ is the distance between the eyes, and *N* corresponds to the number of faces. The baseline NMEs for the TFW test set were reported as 0.036 for the indoor set and 0.404 for the outdoor set [31]. AnyFace++ substantially reduced the error rate, achieving an NME of 0.004 for the indoor and 0.2 for the outdoor set.

### 5.3. Emotion Classification

The accuracy results of emotion classification are in Table 4. When tested on the AffectNet dataset, AnyFace++ performed better than the other models in classifying neutral (81.0%) and happy (92.0%) emotions. In terms of sad (61.6%), surprise (52.5%), and anger (65.4%), the model achieved accuracy levels comparable to the other models. However, the model showed lower accuracy in classifying fear (43.0%) and disgust (32.9%). This discrepancy can be attributed to the significantly lower number of images available in these classes. This problem is more pronounced in our case because this dataset was a small part of the overall dataset.

When tested on the RAF-DB dataset, AnyFace++ achieved an accuracy of 92% for neutral, 94.0% for happy, 83.2% for sad, 83.0% for surprise, and 71.8% for anger, which align with the results of the other models. However, the accuracy for fear (45.9%) and disgust (42.0%) were considerably lower compared to the results of other models. The reasons for this discrepancy are the same as those observed in the case of the AffectNet dataset.

### 5.4. Gender Classification

The accuracy results for gender classification are given in Table 5. When tested on the UTKFace dataset, AnyFace++ achieved an accuracy of 95.4%, comparable to the state-of-the-art result of 96.6%. On the Adience dataset, AnyFace++ performed significantly better than other models by achieving 94.5%. When evaluated on the FairFace test set, AnyFace achieved 93%, close to the baseline accuracy of ResNet-34 (94.4%).

### 5.5. Age Estimation

We employed Mean Absolute Error (MAE) to evaluate the model’s performance in age estimation. MAE represents the average difference between the predicted and actual ages, with lower values indicating better accuracy. The results are shown in Table 6. We conducted tests on the test sets of AgeDB, AFAD, and UTKFace datasets. On the AgeDB dataset, AnyFace++ achieved an MAE of 5.85, comparable to the state-of-the-art result of 5.55 [80]. On the AFAD dataset, AnyFace++ noticeably decreased the MAE of CORAL-CNN [88] from 3.48 to 3.10. AnyFace++ obtained an MAE of 5.17 on the UTKFace dataset, on par with the results of other models.

### 5.6. Qualitative Results

**Figure 3:**
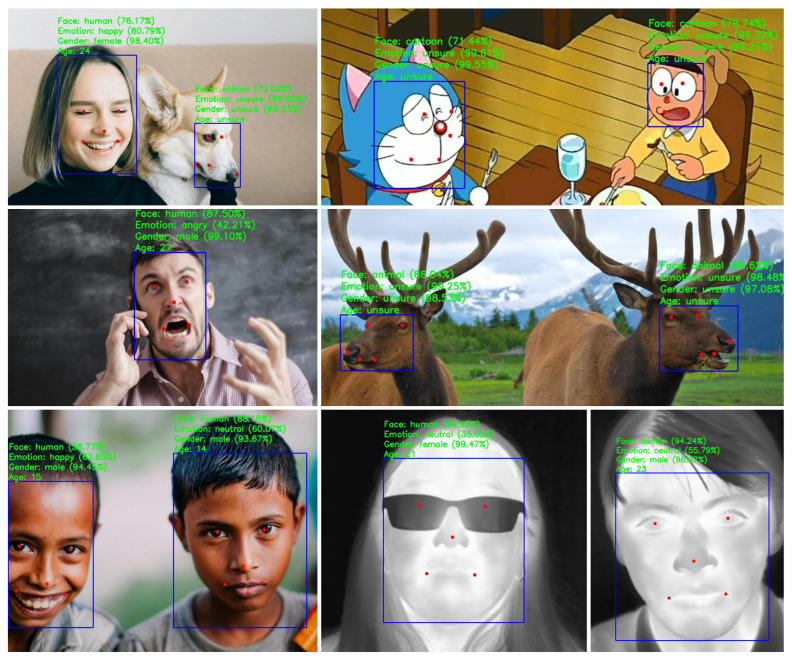
Examples of predictions by the multi-domain, multi-task face AI model, AnyFace++.

Figure 3 presents examples depicting the predictions made by AnyFace++. The model demonstrated successful detection of faces and facial landmarks of animals, different cartoon characters, and humans. It also accurately predicted the age, gender, and emotion of human faces across both visible and thermal domains. The model also correctly classified the age, gender, and emotion of animal and cartoon characters as ”unsure”.

### 5.7. Environmental Impact

AnyFace++ was trained on our private infrastructure, which has a carbon efficiency of 0.432 kg${\mathrm{CO}}_{2}$eq/kWh. The total training time is about 234 h on two A100 SXM4 GPUs. We estimated carbon emissions using the Machine Learning Impact Calculator (https://mlco2.github.io/impact#compute, accessed on 16 April 2024) [91]. The estimated carbon emissions are 80.88 kg${\mathrm{CO}}_{2}$eq, which is equivalent to 40.4 kg of coal being burned. The environmental benefits of AnyFace++ will be during inference because the model performs many tasks in one inference, obviating the need for multiple inferences on the same image.

### 5.8. Limitations

**Figure 4:**
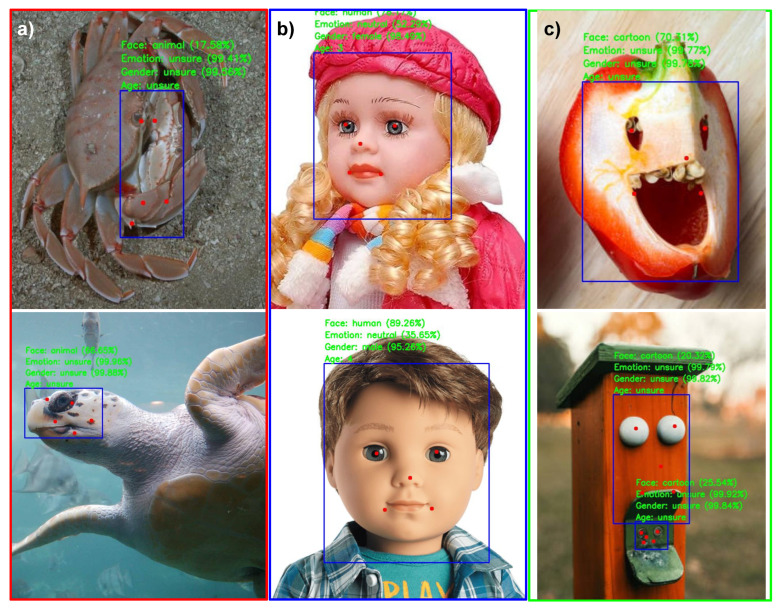
Examples of predictions by AnyFace++: (**a**) underwater animals, (**b**) dolls, and (**c**) facelike objects.

Although AnyFace++ can perform face AI tasks across various domains, such as human, animal, and cartoon faces, it is less accurate in predicting faces and facial landmarks of underwater animals (see Figure 4a) because they were not presented in the training data. In addition, the underwater images pose challenges due to low illumination. We tested the model on the sea turtle faces dataset [92], which comprises 2000 labeled facial images of turtles. The model achieved an $AP_{50}$ score of 0.23. To the best of our knowledge, there are no other labeled datasets of faces for sea animals.

Also, we found that the model tends to predict doll faces as human faces (see Figure 4b). Moreover, the model detects objects that superficially appear to be faces (see Figure 4c). In psychology, this is referred to as face pareidolia, a compelling illusion where people see false faces in everyday objects [93]. The model may experience a similar effect due to the cartoon faces in the training data.

AnyFace++ performs many tasks in one inference cycle, obviating the need for multiple inferences on the same image. However, the model training is resource-intensive because it requires training on large-scale datasets from different domains.

## 6. Conclusions

In this work, we present AnyFace++, an input-agnostic face model designed to execute several face-related tasks concurrently. These tasks include face detection and prediction of facial landmarks across human, animal, and cartoon faces, along with age estimation, gender classification, and emotion recognition exclusive to human faces. The core innovation of AnyFace++ is its multi-task, multi-domain learning framework, which is enhanced by a heterogeneous cost function to handle diverse tasks within a single model effectively.

Despite its advancements, AnyFace++ has some limitations. The model is limited to predicting age, gender, and emotion solely for human faces due to the absence of relevant datasets for cartoon and animal faces. Although it is possible to classify emotions and, in some cases, gender for cartoon characters, the lack of available data prevents training in these areas. For animal faces, while emotion prediction is feasible, predicting age and gender remains limited due to dataset constraints.

The design of the model provides environmental benefits during inference as it performs multiple tasks at once, thereby eliminating the need for multiple inferences on the same image. However, this advantage comes at the cost of increased computational resources during training. The multi-task approach requires extensive resource allocation to process and optimize different tasks and domains simultaneously.

The significance of this study is to propose a universal method that integrates various face-related tasks into a single model, contributing to the development of face analysis technologies. Experimental results substantiate that AnyFace++ delivers results that are on par with the mainstream models which are specifically developed for particular domains. By making the source code and pre-trained AnyFace++ model publicly available, we aim to support further research and innovation in this field, driving forward the capabilities and applications of face analysis systems.

In future work, we aim to use a vision transformer to capture long-range dependencies in images. Additionally, an important improvement will be the addition of face recognition to extend the model’s capabilities beyond face detection and facial landmarks prediction. The model will perform face recognition in multiple domains, making it even more versatile and powerful for real-world applications.

## Figures and Tables

**Table 1:** Statistics of the employed face AI datasets.

Dataset	Domain	Images	Labels	Modality	Resolution	Bbox	Land.	Emotion	Gender	Age
FER [63]	Human	35,887	35,887	Grayscale	48 × 48	crop	−	+	−	−
AffectNet [64]	Human	287,401	287,401	RGB	224 × 224	crop	+	+	−	−
IMDB [65]	Human	285,949	285,949	RGB	various	+	−	−	+	+
UTKFace [66]	Human	23,708	23,708	RGB	200 × 200	crop	+	−	+	+
Adience [67]	Human	13,023	13,023	RGB	various	+	−	−	+	+
MegaAge [68]	Human	41,941	41,941	RGB	178 × 218	add	−	−	−	+
MegaAge-Asian [68]	Human	43,945	43,945	RGB	178 × 219	add	−	−	−	+
AFAD [69]	Human	59,344	59,344	RGB	various	crop	−	−	+	+
AgeDB [70]	Human	16,488	16,488	RGB	various	add	−	−	+	+
FairFace [71]	Human	108,501	108,501	RGB	224 × 224	crop	−	−	+	+
UAGD [72]	Human	11,852	11,852	RGB	various	+	−	−	+	+
FG-NET [73]	Human	1002	1002	RGB	various	+	+	−	−	+
RAF-DB [74]	Human	15,339	15,339	RGB	various	+	+	+	+	+
Wider Face [75]	Human	32,203	393,703	RGB	various	+	+	−	−	−
TFW [31]	Human	9982	16,509	Thermal	464 × 348	+	+	−	+	+
iCartoonFace [76]	Cartoon	60,000	109,810	RGB	various	+	−	−	−	−
AnimalWeb [47]	Animal	19,078	22,451	RGB	various	+	+	−	−	−

+ and − signs indicate the availability of ground-truth labels for each task, *Land.* is facial landmarks, Bbox is facial bounding box, crop means cropped faces, and add means bounding boxes were added using the AnyFace [55] model.

**Table 2:** $AP_{50}$ (%) scores of face detection on the Wider Face dataset (Domain: H-Human, A-Animal, and C-Cartoon).

Method	Validation Set			Test Set			Domain	Params (M)	GFLOPs
	**Easy**	**Medium**	**Hard**	**Easy**	**Medium**	**Hard**			
RetinaFace [30]	96.9	96.1	91.8	96.3	95.6	91.4	H	29.5	37.6
YOLO5Face [28]	96.9	96.0	91.6	94.9	94.3	90.1	H	141.1	88.6
AnyFace [55]	96.7	95.9	91.8	95.2	94.7	90.5	H-A-C	76.7	45.3
AnyFace++ (This Work)	92.9	91.2	83.8	91.6	90.1	82.9	H-A-C	26.6	163.9

**Table 3:** $AP_{50}$ (%) scores of face detection on the test set of AnimalWeb, iCartoonFace, and TFW (Domain: H-Human, A-Animal, and C-Cartoon).

Method	Dataset	AP50	Domain
AnyFace [55]	AnimalWeb	93.6	H-A-C
AnyFace++ (This Work)	AnimalWeb	93.9	H-A-C
ACFD [81]	iCartoonFace	90.9	C
RetinaFace [76]	iCartoonFace	91.0	C
AnyFace [55]	iCartoonFace	91.7	H-A-C
AnyFace++ (This Work)	iCartoonFace	90.8	H-A-C
TFW [31]	TFW (outdoor)	97.3	H
AnyFace [55]	TFW (outdoor)	99.5	H-A-C
AnyFace++ (This Work)	TFW (outdoor)	99.3	H-A-C
TFW [31]	TFW (indoor)	100.0	H
AnyFace [55]	TFW (indoor)	100.0	H-A-C
AnyFace++ (This Work)	TFW (indoor)	100.0	H-A-C

**Table 4:** Accuracy results (%) of emotion classification on the test sets.

Method	Dataset	Neutral	Happy	Sad	Surprise	Fear	Disgust	Anger	Overall
AlexNet + Weighted-Loss [64]	AffectNet	53.3	72.8	61.7	69.9	70.4	68.6	65.8	66.0
POSTER [77]	AffectNet	67.2	89.0	67.0	64.0	64.8	56.0	62.6	67.2
POSTER++ [82]	AffectNet	65.4	89.4	68.0	66.0	64.2	54.4	65.0	67.5
AnyFace++ (This Work)	AffectNet	81.0	92.0	61.6	52.5	43.0	32.9	65.4	61.0
MRE-CNN (VGG-16) [83]	RAF-DB	80.2	88.8	79.9	86.0	60.8	57.5	83.9	76.7
POSTER [77]	RAF-DB	92.4	96.9	91.2	90.3	67.6	75.0	88.9	86.0
POSTER++ [82]	RAF-DB	92.1	97.2	92.9	90.6	68.9	71.9	88.3	86.0
AnyFace++ (This Work)	RAF-DB	92.0	94.0	83.2	83.0	45.9	42.0	71.8	85.0

**Table 5:** Accuracy results of gender classification on the test sets.

Method	Dataset	Acc (%)
CLIP + LP [84]	UTKFace	96.6
AnyFace++ (This Work)	UTKFace	95.4
Hassner et al. [85]	Adience	86.8
PAENet [86]	Adience	89.1
CPG [87]	Adience	89.7
AnyFace++ (This Work)	Adience	94.5
ResNet-34 [71]	FairFace	94.4
AnyFace++ (This Work)	FairFace	93.0

**Table 6:** MAE results of age estimation on the test sets.

Method	Dataset	MAE (Age)
DEX [70]	AgeDB	13.10
MiVOLO-D1 [80]	AgeDB	5.55
AnyFace++ (This Work)	AgeDB	5.85
CORAL-CNN [88]	AFAD	3.48
AnyFace++ (This Work)	AFAD	3.10
CORAL-CNN [88]	UTKFace	5.39
Randomized Bins [89]	UTKFace	4.55
MWR [90]	UTKFace	4.37
AnyFace++ (This Work)	UTKFace	5.17

## Data Availability

Detailed information and relevant links about the data used can be found at https://github.com/IS2AI/AnyFacePP (accessed on 28 February 2023).

## Abbreviations

The following abbreviations are used in this manuscript:

GPUs	Graphics Processing Units
DL	Deep learning
SOTA	State-of-the-art
CNN	Convolutional neural network
DMTL	Deep multi-task learning
MTL	Multi-task learning
DFL	Distribution Focal Loss
CIoU	Complete Intersection over Union
MSE	Mean squared error
NME	Normalized Mean Error
MAE	Mean Absolute Error
